# Supplementary material for: End Sequence Analysis Toolkit (ESAT) expands the extractable information from single-cell RNA-seq data
Source: Genome Res. 2016 Oct;26(10):1397–410. doi: 10.1101/gr.207902.116 (PMC5052061; doi:10.1101/gr.207902.116)
Supplement: Supplemental Material [file supp_gr.207902.116_Supp_Materials.tar.gz › ESAT-esat_v0.1/doc/html/classbroad_1_1pda_1_1gene_1_1_gene_with_isoforms-members.html]

ESAT: Member List


ESAT


- Main Page
- Related Pages
- Packages
- Classes
- Files

- Class List
- Class Index
- Class Hierarchy
- Class Members

All Classes Namespaces Files Functions Variables Enumerator Pages

- broad
- pda
- gene
- GeneWithIsoforms

broad.pda.gene.GeneWithIsoforms Member List

This is the complete list of members for broad.pda.gene.GeneWithIsoforms, including all inherited members.

|  |  |  |
| --- | --- | --- |
| AddAllIsoforms(GeneWithIsoforms overlapper) | broad.pda.gene.GeneWithIsoforms |  |
| AddAllIsoforms(GeneWithIsoforms overlapper, boolean updateMainIsoform) | broad.pda.gene.GeneWithIsoforms |  |
| AddAllIsoNotIncludedforms(GeneWithIsoforms other) | broad.pda.gene.GeneWithIsoforms |  |
| addContainedIsoform(Gene isoform) | broad.pda.gene.GeneWithIsoforms |  |
| AddIsoform(Gene gene) | broad.pda.gene.GeneWithIsoforms |  |
| AddIsoform(Gene gene, boolean updateMainIsoform) | broad.pda.gene.GeneWithIsoforms |  |
| addSuffixToName(String refName) | broad.pda.gene.GeneWithIsoforms |  |
| allIsoformsToBED() | broad.pda.gene.GeneWithIsoforms |  |
| almostContains(Gene other, int exonFudgeFactor) | broad.pda.gene.GeneWithIsoforms |  |
| almostContains(GeneWithIsoforms other, int exonFudgeFactor) | broad.pda.gene.GeneWithIsoforms |  |
| cleanIsoforms() | broad.pda.gene.GeneWithIsoforms |  |
| colapse(int exonFudgeFactor) | broad.pda.gene.GeneWithIsoforms |  |
| constituentIntrons() | broad.pda.gene.GeneWithIsoforms |  |
| constituentIsoform() | broad.pda.gene.GeneWithIsoforms |  |
| dedup() | broad.pda.gene.GeneWithIsoforms |  |
| expandUtrs(Integer utr1, Integer utr2) | broad.pda.gene.GeneWithIsoforms |  |
| findCompatibleGenes(IntervalTree< GeneWithIsoforms > overlapTree, int[] numIntrons) | broad.pda.gene.GeneWithIsoforms |  |
| GeneWithIsoforms(Collection<?extends LightweightGenomicAnnotation > exons) | broad.pda.gene.GeneWithIsoforms |  |
| GeneWithIsoforms(LightweightGenomicAnnotation align) | broad.pda.gene.GeneWithIsoforms |  |
| GeneWithIsoforms(String pslString) | broad.pda.gene.GeneWithIsoforms |  |
| GeneWithIsoforms(String chrString, int start, int end) | broad.pda.gene.GeneWithIsoforms |  |
| GeneWithIsoforms(String chr, int start, int end, String name, Strand orientation, Collection< Annotation > exons) | broad.pda.gene.GeneWithIsoforms |  |
| GeneWithIsoforms(String chr, int start, int end, String name, String orientation, List< Integer > exonsStart, List< Integer > exonsEnd) | broad.pda.gene.GeneWithIsoforms |  |
| GeneWithIsoforms(String chr, int start, int end, String name, String orientation, List< Integer > exonsStart, List< Integer > exonsEnd, int blockStart, int blockEnd) | broad.pda.gene.GeneWithIsoforms |  |
| GeneWithIsoforms(String chr, int start, int end, String name, String orientation, List< Integer > exonsStart, List< Integer > exonsEnd, int blockStart, int blockEnd, String[] extraData) | broad.pda.gene.GeneWithIsoforms |  |
| GeneWithIsoforms(String chr, int start, int end, String name, String orientation, List< Integer > exonsStart, List< Integer > exonsEnd, String[] extraData) | broad.pda.gene.GeneWithIsoforms |  |
| GeneWithIsoforms(Gene gene) | broad.pda.gene.GeneWithIsoforms |  |
| getAllIsoformExonSet() | broad.pda.gene.GeneWithIsoforms |  |
| getAllIsoformIntronSet() | broad.pda.gene.GeneWithIsoforms |  |
| getAllIsoforms() | broad.pda.gene.GeneWithIsoforms |  |
| getAllIsoforms(boolean includeContainingIsoform) | broad.pda.gene.GeneWithIsoforms |  |
| getMerged() | broad.pda.gene.GeneWithIsoforms |  |
| getNumberOfIsoforms() | broad.pda.gene.GeneWithIsoforms |  |
| getScoredExons() | broad.pda.gene.GeneWithIsoforms |  |
| hasOverlapping3PUTR(GeneWithIsoforms other) | broad.pda.gene.GeneWithIsoforms |  |
| hasOverlapping5PUTR(GeneWithIsoforms other) | broad.pda.gene.GeneWithIsoforms |  |
| IsExactOverlappingIsoform(Gene gene) | broad.pda.gene.GeneWithIsoforms |  |
| overlaps(Gene other) | broad.pda.gene.GeneWithIsoforms |  |
| overlapsByGenomicRegion(IntervalTree< GeneWithIsoforms > otherTree, boolean considerOrientation) | broad.pda.gene.GeneWithIsoforms |  |
| overlapsExon(LightweightGenomicAnnotation exon) | broad.pda.gene.GeneWithIsoforms |  |
| overlapsExon(Gene gene) | broad.pda.gene.GeneWithIsoforms |  |
| selectRandIsoSubset(Integer maxIsoPerLoci) | broad.pda.gene.GeneWithIsoforms |  |
| setBedScore(double scr) | broad.pda.gene.GeneWithIsoforms |  |
| setExtraFields(double[] scores) | broad.pda.gene.GeneWithIsoforms |  |
| standardizeOreintation() | broad.pda.gene.GeneWithIsoforms |  |
| toString(String name) | broad.pda.gene.GeneWithIsoforms |  |
| updateScrToBedScore(double score) | broad.pda.gene.GeneWithIsoforms |  |


---

Generated on Fri Aug 15 2014 15:11:00 for ESAT by  

 1.8.7
